# [Retracted] Role of microRNA‑210 in human intervertebral disc degeneration

**DOI:** 10.3892/etm.2023.12210

**Published:** 2023-09-18

**Authors:** Da-Ying Zhang, Zhi-Jian Wang, Yan-Bo Yu, Yong Zhang, Xue-Xue Zhang

Exp Ther Med 11:2349-2354, 2016; DOI: 10.3892/etm.2016.3176

Following the publication of this paper, it was drawn to the Editor’s attention by a concerned reader that, for the flow cytometric experiments shown in Fig. 4A on p. 2352, the images selected for the FasL- and FasL+ experiments were strikingly similar. Moreover, it appeared that various of the data shown in this figure, and western blotting data shown in Fig. 3A, were strikingly similar to data that had appeared in different form in different articles published by different authors at different research institutes.

Owing to the fact that some of the data in the above article had already been published, or were under consideration for publication, prior to its submission to *Experimental and Therapeutic Medicine*, the Editor has decided that this paper should be retracted from the Journal. The authors were asked for an explanation to account for these concerns, but the Editorial Office did not receive a reply. The Editor apologizes to the readership for any inconvenience caused.

